# Call for policy actions based on evidence from the Policy Evaluation Network

**DOI:** 10.1093/eurpub/ckac146

**Published:** 2022-11-29

**Authors:** Wolfgang Ahrens, Jeroen Lakerveld, Catherine B Woods

**Affiliations:** Leibniz Institute for Prevention Research and Epidemiology — BIPS, Bremen, Germany; Department of Epidemiology and Data Science, Amsterdam UMC, VU University Amsterdam, Amsterdam Public Health Research Institute, Amsterdam, The Netherlands; Upstream Team, Amsterdam UMC, VU University Amsterdam, Amsterdam, The Netherlands; Department of Physical Education and Sport Sciences, Physical Activity for Health Research Centre, Health Research Institute, University of Limerick, Limerick, Ireland

Chronic diseases caused by physical inactivity and poor nutrition are the leading cause of disability and death in Europe. Cardiovascular diseases, diabetes, cancer and respiratory diseases accounting for 77% of the burden of disease and almost 86% of premature mortality,^1^^,^^2^ and these diseases are still on the rise. Physical inactivity, sedentary behaviours, excessive consumption of energy, saturated fatty acids, trans-fatty acids, sugar and salt, as well as low consumption of vegetables, fruits and whole grains are behavioural determinants of chronic diseases and deserve the highest priority for counteractive measures.^3^ In addition to the role of these risk factors as drivers of non-communicable diseases, our understanding of their impact on the burden of communicable diseases such as COVID is only beginning to emerge. We have learnt that the context in which the above-mentioned health-related behaviours take place, i.e. in the social, political and built environmental domains, has to be addressed to effectively change behaviours and prevent or reduce diseases downstream. This requires a systems perspective to achieve the desired health outcomes on the population level.^4^^,^^5^ Here policy comes into play.

Successful policy actions to address physical inactivity, unhealthy diets and sedentary behaviour have the potential to influence the health and well-being of an entire population. The policy is about changing systems not people and involves a mix of decisions and actions. Public policies include processes activated across multiple sectors to achieve specific outcomes, a delicate balance between what is achievable, what is evidence informed and what has political will or ‘buy in’. The UN Sustainable Development Goals’ (https://sustainabledevelopment.un.org/), the Global Action Plan on Physical Activity^6^ and WHO’s executive summary of the UN decade of action on nutrition^7^ highlight the need to move beyond *individual* behaviour change to broader *policy and system* approaches, focusing not only on health but also on sustainability. Yet systematic research on the assessment or evaluation of policy interventions across Europe is sparse. Consequently, limited information on the merit, worth or utility of policy interventions is available with little guidance or recommendations on how to address this gap in our knowledge.

As part of the Joint Programming Initiative on a Healthy Diet for a Healthy Life (JPI HDHL), 28 research institutes from 7 European countries and New Zealand combined their expertise to form a Policy Evaluation Network (PEN). PEN’s vision is to provide Europe with tools to identify, evaluate and benchmark policies designed to directly or indirectly address physical inactivity, unhealthy diets and sedentary behaviour while accounting for existing health inequities.

This journal supplement comprises 13 manuscripts, output from the PEN project. Topics examined include new theoretical models to advance our understanding of the policy process and its evaluation. The supplement provides an overview of priority public policies and policy areas most likely to sustainably reduce physical inactivity, unhealthy diets and sedentary behaviour. It explores the first steps in a bespoke policy monitoring and surveillance system for Europe and a refinement of our knowledge of appropriate research designs and methods for the quantification of policy impact. PEN scientists illustrate, in this issue, how best to evaluate the implementation and impact of policy in order to yield the best results for a healthy life of European citizens. Importantly, PEN has provided recommendations on equity and diversity to ensure that policy actions are inclusive as opposed to exclusive, are relevant to the changing landscape of Europe and are culturally sensitive.

Through this supplement, we make a call to action. A call to governments and policymakers from national and local levels to use the newly acquired knowledge to develop, implement and evaluate strong, comprehensive policy solutions that are sustainable, equitable and address the current health challenges effectively. A call to funding agencies at European, national and local levels to continue to prioritize and fund policy research, to build on the work of PEN which has really only begun to understand the potential policy impact on human behaviours and environments. A call to policy researchers to move beyond peer-reviewed publications and to translate their findings into relevant and meaningful advice and advocacy documents for policymakers to use to advance policy action to promote health-for-all citizens across Europe.
